# Engineering an in vivo charging station for CAR-redirected invariant natural killer T cells to enhance cancer therapy

**DOI:** 10.21203/rs.3.rs-6215345/v1

**Published:** 2025-04-10

**Authors:** Song Li, Yan-Ruide Li, Haochen Nan, Zeyang Liu, Ying Fang, Yichen Zhu, Zibai Lyu, Zhengyao Shao, Enbo Zhu, Bo zhang, Youcheng Yang, Xinyuan Shen, Yuning Chen, Tzung Hsiai, Lili Yang

**Affiliations:** UCLA; UCLA; University of California, Los Angeles; University of California, Los Angeles; UCLA; UCLA; University of California, Los Angeles; UCSD; UCLA; University of California, Los Angeles; UCLA; University of California, Los Angeles; University of California, Los Angeles; University of California Los Angeles; University of California, Los Angeles

**Keywords:** Invariant natural killer T (iNKT) cells, chimeric antigen receptor (CAR), iNKT cell-targeted Microparticle Recruitment and Activation System (iMRAS), implanted biomimetic scaffold, cancer immunotherapy

## Abstract

Invariant natural killer T (iNKT) cells are a distinct subset of T lymphocytes that possess unique properties making them highly suitable for addressing the challenges of solid tumor immunotherapy. Unlike conventional T cells, which are restricted by polymorphic major histocompatibility complex (MHC) molecules and recognize peptide antigens, iNKT cells are restricted by the non-polymorphic CD1d molecule and respond to lipid antigens. Chimeric antigen receptor (CAR)-redirected iNKT (CAR-iNKT) cells represent a significant advancement in cancer immunotherapy. However, optimizing sustained activation and long-term persistence of CAR-iNKT cells remains a critical need for effective solid tumor treatment. To address these limitations, we develop the iNKT cell-targeted microparticle recruitment and activation system (iMRAS), a biomimetic platform designed to enhance iNKT cell functionality through localized immunostimulation in vivo. This biomimetic platform is designed to function as an in vivo “charging station” containing chemotactic and activation signals for the recruitment, activation, and expansion of CAR-iNKT cells, leading to more effective tumor killing and longer persistence of CAR-iNKT cells, as demonstrated in the therapy of lymphoma and melanoma. Through its biomimetic design and localized immunostimulatory effects, iMRAS helps overcome the limitations of current therapies for solid tumors, establishing a robust platform for enhancing systemic CAR-iNKT cell-mediated immunotherapy.

## Introduction

Human invariant natural killer T (iNKT) cells are a distinct subset of T lymphocytes that function at the interface of innate and adaptive immunity^1,2^. Unlike conventional T cells, which are restricted by polymorphic major histocompatibility complex (MHC) molecules and recognize peptide antigens, iNKT cells are restricted by the non-polymorphic CD1d molecule and respond to lipid antigens. Despite their low frequency among total T cells, iNKT cells possess remarkable immunomodulatory and antitumor capabilities, characterized by their rapid response to activation and the secretion of a wide range of cytokines^3–5^. These unique properties enable iNKT cells to influence both immune activation and regulation in various contexts, including cancer. Increasing evidence has shown that higher iNKT cell frequency and functionality are positively correlated with improved clinical outcomes in various malignancies including colorectal cancer and head and neck squamous cell carcinomas^6–9^. Their distinct biology and potent immune responses position iNKT cells as a promising platform for innovative approaches in cancer immunotherapy, including adoptive cell therapy and pharmacological activation.

Building on their unique immunological features, iNKT cells have emerged as an attractive platform for engineered cellular immunotherapy. Chimeric antigen receptor (CAR)-redirected iNKT (CAR-iNKT) cells represent a significant advancement in cancer immunotherapy. By integrating a tumor-targeting CAR with the natural tumor recognition mechanisms of iNKT cells, CAR-iNKT cells exhibit enhanced antitumor efficacy through multiple pathways^10–12^. Besides utilizing the CAR construct, CAR-iNKT cells leverage their inherent CD1d restriction and natural killer receptors (NKRs) to recognize and attack tumors^13,14^.

This combinatorial targeting enables CAR-iNKT cells to exert potent cytotoxicity, particularly in challenging tumor microenvironments (TMEs) characterized by immune suppression^10–12^. Importantly, CAR-iNKT cells are adept at modulating the TME by targeting and reducing the immunosuppressive activity of tumor-associated macrophages (TAMs) and myeloid-derived suppressor cells (MDSCs), thereby creating a more favorable environment for effective immune responses^10–12^.

Preclinical and clinical studies have underscored the robust therapeutic potential of CAR-iNKT cells across a range of malignancies, including B cell maliganancies^15,16^, multiple myeloma^11,17,18^, acute lymphoblastic leukemia (ALL)^19^, neuroblastoma^20–22^, liver cancer^23^, lung cancer^24^, and ovarian cancer^25^. A recent clinical trial evaluated GD2-targeting CAR-iNKT cells in pediatric patients with neuroblastoma, demonstrating both potent antitumor efficacy and a high safety profile^21,22^. Notably, this trial overexpressed IL-15 in the CAR-iNKT cells, which enhanced their *in vivo* persistence and antitumor function^21,22^. Additionally, the innate-like properties and restricted TCR repertoire of iNKT cells make them a promising allogeneic cell source for off-the-shelf immunotherapy, reducing the risks of graft-versus-host disease while maintaining potent antitumor activity. Compared to conventional CAR-T cells, CAR-iNKT cells are associated with lower risks of adverse effects such as cytokine release syndrome (CRS), making them a safer alternative for adoptive cell therapy^21,22^. These studies collectively highlight the promise of CAR-iNKT cells as a versatile and effective therapeutic option, especially for tumors with immunosuppressive microenvironments.

Despite these promising results, several challenges remain in optimizing CAR-iNKT cell therapy, particularly for solid tumors. Like conventional CAR-T cells, CAR-iNKT cells face obstacles in efficiently trafficking and infiltrating solid tumors, maintaining sustained activation, and achieving long-term persistence. Strategies such as the administration of iNKT cell agonists like α-galactosylceramide (αGC) or αGC-pulsed dendritic cells (DCs) have been explored to enhance iNKT cell activation and recruitment^3,26,27^. However, these approaches are limited by short half-lives and reduced efficacy over time, leading to inconsistent activation and diminished therapeutic impact^4^. Additionally, solid tumors often present physical and immunosuppressive barriers that hinder the infiltration and function of CAR-iNKT cells^28^. Therefore, innovative strategies are urgently needed to overcome these limitations and fully harness the therapeutic potential of CAR-iNKT cells in solid tumor settings.

**Figure 1:**
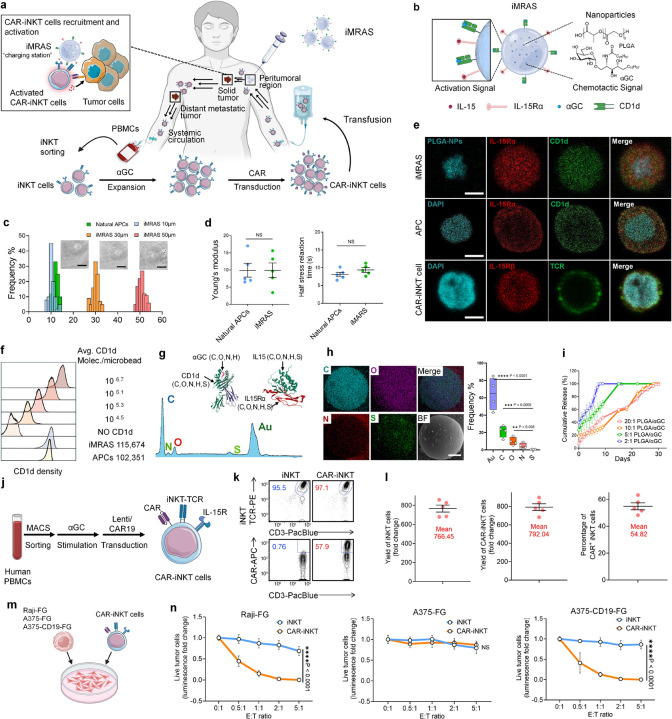
Development, characterization, and functional evaluation of the iMRAS and CAR-iNKT cells. **a**, Schematic of iMRAS-enhanced CAR-iNKT cell therapy. iMRAS promotes CAR-iNKT cell recruitment, activation, and tumor infiltration while enhancing systemic immune activation to improve therapeutic efficacy against solid tumors. **b**, Schematic representation of the iMRAS (invariant NKT-targeted microparticle activation and recruitment system). iMRAS consists of alginate-based microparticles encapsulating nanoparticles for sustained release of α-GalCer (αGC) and IL-15. The surface of iMRAS is functionalized with CD1d presenting αGC and IL-15Rα/IL-15 complexes to recruit and activate iNKT cells. **c**, Size distribution of iMRAS microparticles showing tunable diameters that can closely mimic the size of natural antigen-presenting cells (APCs). Insets: representative bright-field microscopy images of iMRAS microparticles. **d**, Mechanical properties of iMRAS, including stiffness and viscoelasticity, demonstrating biomimicry of natural APCs. No significant differences (NS) were observed compared to natural APCs (n = 5). **e**, Fluorescent imaging confirming the morphological and antigenic biomimicry of iMRAS. Uniform surface distribution of CD1d and IL-15Rα/IL-15 complexes on iMRAS microparticles was observed. Scale bar: 10 μm. **f**, Quantification of antibody density on iMRAS microparticles using a bead-calibration system, revealing comparable antibody numbers on iMRAS and dendritic cells (DCs) **g**, Surface elemental composition analysis of iMRAS via spectrum analysis, indicating the presence of nitrogen (N), suggesting uniform distribution of functional antibodies on the surface. **h**, Elemental mapping of iMRAS microparticles further confirms the homogeneous distribution of nitrogen and sulfur, showing the uniform coating of functional molecules (n = 4) **i**, Drug release kinetics of αGC from iMRAS microparticles under varying PLGA-to-αGC mass ratios, demonstrating tunable release profiles (n = 4). **j**, Diagram depicting the process of generating CAR-iNKT cells from human PBMCs, including sorting, transduction, and stimulation steps. **k**, Flow cytometry analysis of the generated CAR-iNKT cells. The cells exhibited 97.1% TCR expression and 57.9% CAR expression. **l**, Proliferation and expansion of iNKT cells after *in vitro* culture and CAR transduction, with high yields achieved (n = 5). **m**, Diagram illustrating the co-culture experiment of CAR-iNKT cells with tumor cells to assess cytotoxicity. **n**, Cytotoxic activity of CAR-iNKT cells against various tumor cell lines (Raj-FG, A375-FG, and A375-CD19-FG). CAR-iNKT cells demonstrated superior tumor-killing efficacy compared to non-CAR-expressing iNKT cells, indicating their potential for adoptive cell therapy (n = 4). Data are presented as the mean ± s.e.m. NS, not significant; *P < 0.05, **P < 0.01, ***P < 0.001, ****P < 0.0001, by Student’s t test (**d**), one-way ANOVA (**h**), or two-way ANOVA (**n**).

To address these limitations, we have developed the iNKT cell-targeted microparticle recruitment and activation system (iMRAS), a biomimetic platform that integrates fine-tuned mechanical properties, ligand density, and drug-encapsulated nanoparticles to provide both localized and systemic immunomodulatory effects (Fig. 1a). We demonstrated that iMRAS enhances the recruitment and activation of CAR-iNKT cells *in vitro* and *in vivo*, leading to improved anti-tumor efficacy. The implanted iMRAS can act as a “charging station” for circulating iNKT cell to enable the recruitment, activation, and expansion of CAR-iNKT cells with stronger tumor killing capability and longer persistence in the body in a systemic manner. Through its biomimetic design and localized immunostimulatory effects, iMRAS is expected to overcome the limitations of current therapies for solid tumors, establishing a robust platform for enhancing iNKT cell-mediated immunotherapy. By leveraging the unique design of iMRAS, this work aims to develop a scalable platform to evaluate its impact on CAR-iNKT cell recruitment, activation, tumor-killing efficiency, and systemic immune modulation, ultimately addressing critical challenges in cancer immunotherapy.

## Results

### Development and Characterization of iMRAS as Biomimetic Scaffold for CAR-iNKT Cell Recruitment and Activation

To enhance the recruitment and activation of human CAR-iNKT cells for improved immunotherapy, we developed an innovative biomimetic platform termed iMRAS (Fig. 1b). iMRAS was engineered using alginate hydrogels embedded with poly(lactic-co-glycolic acid) (PLGA) nanoparticles loaded with αGC^29^, a potent glycolipid agonist that binds CD1d molecules to induce iNKT cell activation and antitumor immunity. αGC within iMRAS serves as a chemoattractant, agonist, and sustained-release immunomodulator, continuously diffusing to recruit and activate iNKT cells. While soluble αGC can activate iNKT cells, its rapid clearance and systemic diffusion often result in suboptimal activation and limited therapeutic efficacy^30^. To address this challenge, we conjugated CD1d molecules presenting αGC on the surface of iMRAS^31^. In addition, to mimic the trans-presentation of IL-15 by natural antigen-presenting cells (APCs), we conjugated IL15Rα onto iMRAS surface to load and present IL15 through the IL15/IL15Rα transpresentation complexes (IL15Tx), which are essential for the activation, maturation, and cytotoxic function of iNKT cells. This iMRAS design is to achieve the recruitment of CAR-iNKT cells, sustained CAR-iNKT cell activation, and enhanced antitumor capacity *in vivo*.

Using microfluidic technology, we precisely tuned the size of iMRAS microparticles to diameters of 8–15 μm, closely resembling the dimensions of natural APCs (Fig. 1c). This size optimization enhances their biophysical compatibility with immune cells, improving interaction efficiency. The microfluidic approach allows for high reproducibility, uniform particle size distribution, and scalability, making it a highly efficient method for generating biomimetic microparticles (**Supplementary Fig. 1**). We used atomic force microscopy (AFM) indentation measurements to quantify the stiffness and viscoelastic properties of iMRAS, revealing values comparable to those of natural APCs (Fig. 1d; **Supplementary Fig. 2a**). Additionally, rheometry analysis was performed to assess the bulk mechanical properties of iMRAS in the form of 2D gel discs, with comparative analysis against elastomeric polydimethylsiloxane (PDMS). The loss factor analysis indicated that iMRAS exhibits a highly viscoelastic nature compared to purely elastic materials, further confirming its biomimetic properties and functional relevance for iNKT cell activation (**Supplementary Fig. 2b, c**). Furthermore, swelling and weight retention tests demonstrated that iMRAS hydrogels maintained stable physical properties over a two-week period, exhibiting no significant swelling or degradation (**Supplementary Fig. 2d**). Accumulating evidence suggests that biomechanical properties play a crucial role in T cell receptor (TCR) activation, influencing signal strength, T cell fate, and immune response efficiency^32–35^. The stiffness and viscoelasticity of iMRAS were precisely tuned by adjusting the concentration of calcium ion crosslinkers and the molecular weight of alginate, enabling fine control over its biomechanical properties^36^. Such biomechanical cues contribute to improved immune cell recruitment and enhanced therapeutic efficacy of CAR-iNKT cell therapies.

To provide activation signals for iNKT cells, the iMRAS was functionalized with CD1d/αGC and IL-15Tx complexes. Fluorescence imaging confirmed the uniform distribution of these molecules on the particle surface while also demonstrating the presence of encapsulated nanoparticles with chemotactic signals (Fig. 1e). Compared to APCs, iMRAS exhibits a high degree of biomimicry, incorporating both recruitment and activation signals for iNKT cells (Fig. 1f). Flow cytometry analysis demonstrated that the density of CD1d molecules on the iMRAS surface was comparable to that of natural dendritic cells based on the calibration beads with known antibody densities (Fig. 1g). Elemental analysis using energy-dispersive X-ray spectroscopy revealed the presence of nitrogen and sulfur on the particle surface, indicative of functional molecule coating (Fig. 1h). From the distribution of nitrogen and sulfur signals, a uniform modification pattern was observed, representing the homogeneous ligand decoration across the iMRAS surface.

### Fabrication and Characterization of PLGA Nanoparticles for Sustained Release of αGC

To optimize the sustained release of αGC and enhance its therapeutic efficacy, we fabricated PLGA nanoparticles using a solvent evaporation method combined with ultrasonic homogenization at a fixed frequency of 20 kHz (**Supplementary Fig. 3a**). This approach allowed for precise control over nanoparticle size and drug encapsulation efficiency, crucial for achieving a controlled and prolonged αGC release profile.

Controlling nanoparticle size is crucial, as it directly affects the degradation rate of PLGA and subsequently modulates drug release kinetics. Higher ultrasonic homogenization amplitude (50%) resulted in smaller nanoparticles (~200 nm), whereas lower amplitude (10%) produced larger nanoparticles (~600 nm), with a direct correlation observed between amplitude and size (**Supplementary Fig. 3b**). Increasing PLGA concentration from 1 mg/mL to 40 mg/mL resulted in a size increase from ~100 nm to ~250 nm (**Supplementary Fig. 3c**). The choice of organic solvent significantly affected nanoparticle size, with nanoparticles prepared using dichloromethane being smaller compared to those prepared using acetonitrile and tetrahydrofuran (**Supplementary Fig. 3d**). The efficiency of αGC encapsulation within PLGA nanoparticles was assessed under various conditions. Encapsulation efficiency of the formulation increased with the PLGA: αGC ratio, with 20:1 yielding the highest efficiency and 2:1 the lowest (**Supplementary Fig. 3e**). Higher molecular weight PLGA (50 kDa) exhibited the highest encapsulation efficiency, while lower molecular weight PLGA (15 kDa) resulted in lower efficiency (**Supplementary Fig. 3f**). This difference is likely due to the increased polymer chain entanglement in higher molecular weight PLGA, which enhances drug retention within the nanoparticle matrix.

Drug release kinetics was analyzed to determine the optimal formulation for sustained αGC delivery. Cumulative release profiles demonstrated a controlled release over 30 days, with an initial rapid release within the first 5 days followed by a sustained release phase, which varied depending on the PLGA composition (Fig. 1i). The ratio of lactic acid (LA) to glycolic acid (GA) in PLGA copolymer played a significant role in modulating drug release kinetics, with higher LA content leading to slower degradation and extended drug release (**Supplementary Fig. 3g**). Additionally, the release kinetics was also dependent on the PLGA-to-αGC ratio, with the 20:1 formulation exhibiting the slowest release rate, while the 2:1 formulation demonstrated the fastest αGC release, suggesting that polymer composition critically influences drug availability over time. Based on optimal encapsulation efficiency and drug release kinetics, a PLGA-to-αGC ratio of 20:1 and an LA:GA ratio of 1:1 were selected for subsequent experiments.

### Generation of Human CAR-iNKT Cells with High Yield, Purity, and Tumor-Killing Efficacy

We successfully generated human CD19-targeting CAR-iNKT cells from healthy donor peripheral blood mononuclear cells (PBMCs). iNKT cells were isolated using Anti-iNKT microbeads and cultured with αGC stimulation; subsequently, they were transduced with a lentiviral vector encoding the CD19-targeting CAR (Fig. 1j). Utilizing this approach, we achieved a high yield of CAR-iNKT cells (> 700-fold increase), high purity (> 95%), and significant robustness, with testing conducted across more than five donors (Fig. 1k, l). We routinely attained a high CAR engineering percentage (> 50%) in CAR-iNKT cells (Fig. 1k, l).

To evaluate the tumor-targeting efficacy and mechanism of action (MOA) of CAR-iNKT cells, we employed three tumor cell lines: the CD19^+^ human lymphoma line Raji, the CD19- human melanoma line A375, and an engineered A375 line overexpressing CD19 (A375-CD19). All three tumor cell lines were further modified to express firefly luciferase and enhanced green fluorescent protein (FG), enabling real-time monitoring of tumor cells via flow cytometry and bioluminescence assays (Fig. 1m).

*In vitro* tumor cell killing assays demonstrated that CAR-iNKT cells exhibited potent cytotoxicity against CD19^+^ tumor cells compared to non-CAR-engineered iNKT cells (Fig. 1n). In contrast, no significant difference in cytotoxicity was observed between CAR-iNKT and iNKT cells when targeting CD19^−^ tumor cells (Fig. 1n). Overall, CAR-iNKT cells can be generated with high yield, high purity, and robust antitumor efficacy, making them suitable for subsequent experimental applications.

### *In vitro* iMRAS-Driven CAR-iNKT Cell Activation and Tumor Killing Efficacy.

**Figure 2:**
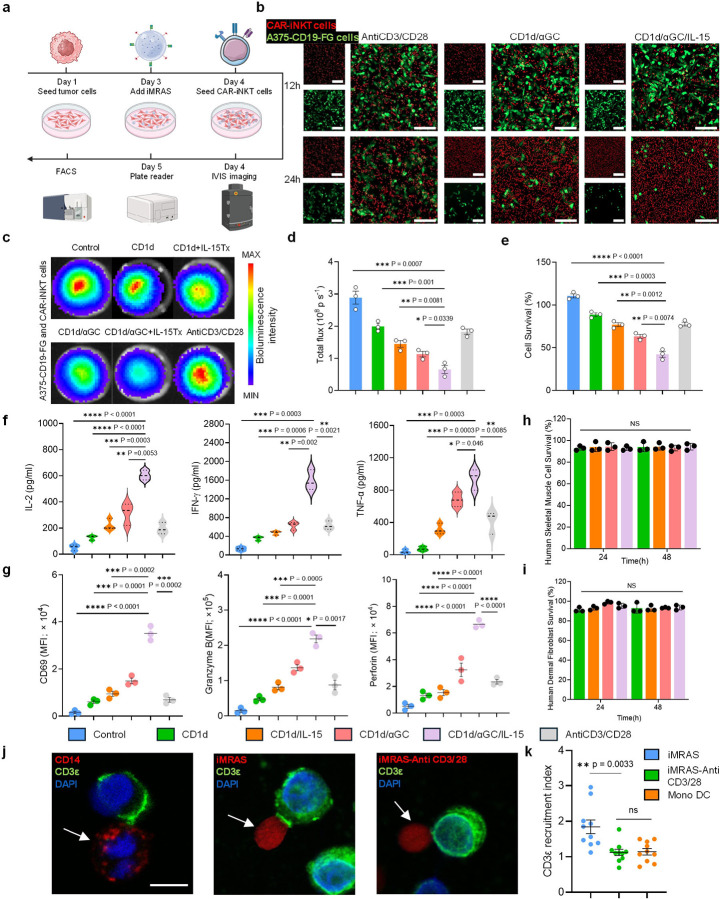
*In vitro* functional validation of iMRAS in activating CAR-iNKT cells and inducing tumor cell cytotoxicity. **a**, Schematic representation of the experimental timeline for in vitro assessment of iMRAS functionality. On Day 1, tumor cells (A375-CD19-FG) were seeded, followed by Day 3 iMRAS addition and Day 4 CAR-iNKT cell seeding. On Day 5, various functional assays were performed, including fluorescence imaging, bioluminescence imaging, flow cytometry, and ELISA to evaluate CAR-iNKT cell activation, cytokine secretion, and tumor cell killing efficiency (n = 3). **b**, Fluorescent imaging of tumor cell viability at 12 and 24 hours under different conditions: bare iMRAS (no surface modification), CD1d/αGC/IL-15 conjugated iMRAS, and anti-CD3/CD28 conjugated iMRAS. Red fluorescence indicates tumor cell survival, while green fluorescence indicates dead tumor cells (n = 3). Scale bar: 50 μm. **c**, Bioluminescence imaging of A375-CD19-FG tumor cells in six experimental groups, representing different iMRAS modifications. Images display differences in bioluminescence intensity corresponding to tumor cell viability (n = 3). **d**, Quantification of bioluminescence flux and tumor cell viability in different groups, showing the enhanced cytotoxic effects of CD1d/αGC/IL-15 conjugated iMRAS (n = 3). **e**, Tumor cell survival rates (%) across different experimental groups at 24 hours, with CD1d/αGC/IL-15 conjugated iMRAS demonstrating significantly reduced survival (n = 3). **f**, ELISA results showing the release of cytokines (i.e., IL-2, IFN-γ, and TNF-α) by CAR-iNKT cells in response to different iMRAS modifications (n = 3). **g**, Flow cytometry analysis of CAR-iNKT cell activation, assessing surface marker expression (i.e., CD69), and effector molecules (i.e., Granzyme B and Perforin) (n = 3). **h** and **i**, Evaluation of iMRAS cytotoxicity against non-tumor cells. Human dermal fibroblasts (HDFs) and human skeletal muscle cells (HSMCs) were co-cultured with iMRAS. No significant cytotoxicity was observed at 24 or 48 hours, demonstrating the biocompatibility of iMRAS (n = 3). **j**, Representative immunofluorescence images show CD3ε-staining in CAR-iNKT cells (green) that interacted with MoDCs labeled with anti-CD14 (red; left panel), iMRAS (red; middle panel) or iMRAS-anti-CD3/CD28 (red; right panel), indicated by white arrows. Scale bar: 10 μm. **k**, CD3ε recruitment index in CAR-iNKT cells across different conditions. CD3ε recruitment in the iMRAS group increase significantly. Data are presented as the mean ± s.e.m. NS, not significant; *P < 0.05, **P < 0.01, ***P < 0.001, ****P < 0.0001, by one-way ANOVA (**d-i** and **k**).

To evaluate the functionality and safety of iMRAS *in vitro*, we conducted co-culture experiments involving CAR-iNKT cells, iMRAS microparticles, and tumor cells. The experimental setup included six groups: (1) control (unmodified iMRAS), (2) iMRAS conjugated with CD1d only, (3) iMRAS conjugated with CD1d and IL-15Tx, (4) iMRAS conjugated with CD1d/αGC, (5) iMRAS conjugated with CD1d/αGC and IL-15Tx, and (6) iMRAS conjugated with anti-CD3/CD28 antibodies. Notably, none of these groups included PLGA nanoparticles loaded with αGC, as this experiment focused primarily on activation rather than recruitment. The tumor cells used in these experiments were CD19-expressing A375 cells. Tumor cells were seeded on Day 1, followed by the addition of iMRAS on Day 3 and CAR-iNKT cells on Day 4. Flow cytometry, bioluminescence imaging, and plate reader assays were performed at different time points to assess CAR-iNKT cell functionality (Fig. 2a).

Fluorescent microscopy and bioluminescence imaging revealed significantly enhanced tumor cell killing in the group treated with iMRAS conjugated with CD1d/αGC+IL-15Tx compared to other conjugation strategies, including control and CD1d-only treatments (Fig. 2b, c). Quantitative analysis of bioluminescence flux confirmed these findings, demonstrating the most tumor cell death in the iMRAS conjugated with CD1d/αGC+IL-15Tx group (Fig. 2d, e). Further, ELISA assays showed that CAR-iNKT cells co-cultured with iMRAS conjugated with CD1d/αGC+IL-15Tx secreted significantly higher levels of effector cytokines, including IL-2, IFN-γ, and TNF-α, compared to other experimental groups (Fig. 2f). Flow cytometry analysis revealed increased expression of activation markers (CD69) and cytotoxic molecules (granzyme B and perforin) in CAR-iNKT cells stimulated by iMRAS conjugated with CD1d/αGC+IL-15Tx (Fig. 2g). These findings indicate that iMRAS effectively activates CAR-iNKT cells to exert potent antitumor effects. To evaluate the biosafety of iMRAS, human dermal fibroblasts and human skeletal muscle cells were co-cultured with iMRAS microparticles under identical conditions. No significant cytotoxic effects were observed in either cell type after 24 or 48 hours of incubation (Fig. 2h, i). Confocal microscopy imaging revealed CD3ε recruitment in CAR-iNKT cells co-cultured with iMRAS, further supporting its role in enhancing immune synapse formation and T cell activation (Fig. 2j). Quantitative analysis showed that iMRAS significantly enhanced CD3ε recruitment compared to control groups, including iMRAS modified with anti-CD3/CD28 and monocyte-derived dendritic cells (MoDCs) pulsed with αGC (Fig. 2k). Notably, MoDCs represent the gold standard for iNKT cell activation, while anti-CD3/CD28 stimulation provides a broad, non-specific activation signal. The superior performance of iMRAS in CD3ε recruitment highlights its ability to provide a targeted and physiologically relevant activation strategy for CAR-iNKT cells.

### Single Cell Transcriptomic Analysis of CAR-iNKT Activation by iMRAS

**Figure 3:**
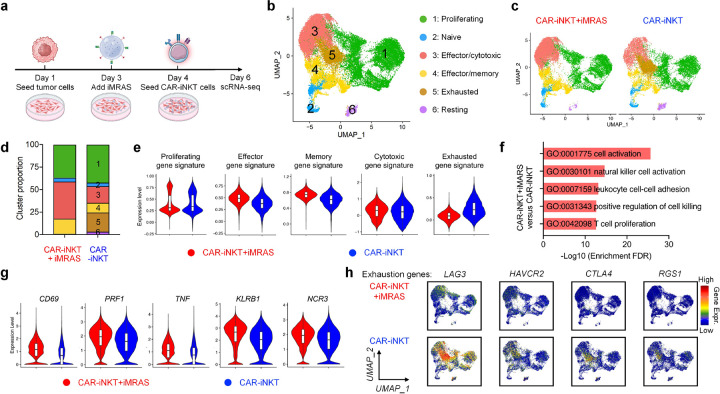
scRNA-seq reveals that iMRAS enhances CAR-iNKT cell activation and memory formation, and reduces exhaustion compared to αGC-pulsed MoDCs. **a**, Experimental design for scRNA-seq study, where CAR-iNKT cells were co-cultured with tumor cells, with or without the activation of iMRAS. **b**, UMAP clustering of CAR-iNKT cells identifies distinct functional populations, including proliferating, naïve, effector/cytotoxic, effector/memory, exhausted, and resting cells. **c**, UMAP projection comparing CAR-iNKT cells activated with or without iMRAS. **d**, Proportional distribution of CAR-iNKT cell populations across conditions. **e**, Violin plots showing expression of proliferating, effector, memory, cytotoxic, and exhausted gene signatures in CAR-iNKT cells from both conditions. **f**, Gene ontology (GO) enrichment analysis highlighting pathways significantly upregulated in the iMRAS-traeted CAR-iNKT cells. **g**, Violin plots showing expression of the indicated genes in CAR-iNKT cells from both conditions. **h**, UMAP plots showing the expression of exhaustion-related genes in CAR-iNKT cells from both conditions. Cells collected from three repeated experiments were combined for analyses. In the violin plots (**e**and **g**), box and whisker plots exhibit the minimum, lower quartile, median, upper quartile and maximum expression levels of each type of cell.

To investigate the genomic and molecular characteristics of CAR-iNKT cells with or without iMRAS activation, we conducted an *in vitro* tumor cell killing assay. In this assay, A375-CD19 melanoma tumor cells were co-cultured with CAR-iNKT cells, both with and without iMRAS, for a duration of three days. Post co-culture, the CAR-iNKT cells were collected and subjected to single-cell RNA sequencing (scRNA-seq) analysis (Fig. 3a).

Uniform manifold approximation and projection (UMAP) analysis of combined two samples revealed the formation of 6 major cell clusters (Fig. 3b). The signature gene profiling and Gene Set Enrichment Analysis (GSEA) identified cluster 1 cells as proliferating cells, cluster 2 cells as naïve cells, cluster 3 cells as effector/cytotoxic cells, cluster 4 cells as effector/memory cells, cluster 5 cells as exhausted cells, and cluster 6 cells as resting cells (**Supplementary Fig. 4 and 5**)^11,37^.

Compared to CAR-iNKT cells alone, iMRAS-treated CAR-iNKT cells demonstrated an increase in effector/cytotoxic and effector/memory cell populations, along with a significant reduction in exhausted cells (Fig. 3c, d). Gene signature analysis further corroborated the enhanced activation state of iMRAS-treated CAR-iNKT cells, evidenced by elevated expression of effector, memory, and cytotoxic gene signatures, coupled with reduced expression of exhaustion gene signatures (Fig. 3e). Gene Ontology (GO) enrichment analysis highlighted significant upregulation of pathways related to cell activation, NK cell activation, leukocyte adhesion, and T cell proliferation in iMRAS-treated CAR-iNKT cells (Fig. 3f). Gene expression analysis revealed higher levels of activation, cytotoxicity, and memory markers (e.g., *CD69, PRF1, TNF, KLRB1*, and *NCR3*) in iMRAS-activated CAR-iNKT cells (Fig. 3g), correlating with their enhanced tumor-killing potential (Fig. 2). Notably, exhaustion marker-encoding genes (e.g., *LAG3, HAVCR2, CTLA4, RGS1*) were lower in iMRAS-treated CAR-iNKT cells (Fig. 3h), suggesting reduced exhaustion and sustained immune function^38–40^. These findings validate iMRAS as a superior immunostimulatory platform that enhances CAR-iNKT cell activation, persistence, and anti-tumor efficacy while reducing exhaustion, further supporting the role of mechanical and biochemical cues of iMRAS in modulating CAR-iNKT cell responses for improved therapeutic outcomes.

### *In vivo* Safety and Biodistribution of iMRAS

**Figure 4:**
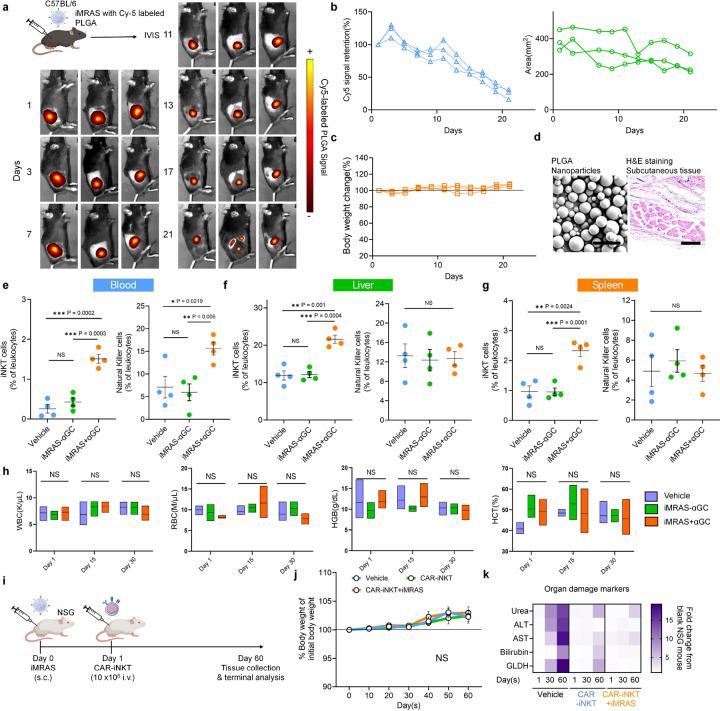
Safety evaluation of the iMRAS system *in vivo*. **a**, *In vivo*tracking of mouse-version iMRAS using Cy5-labeled PLGA microparticles in C57BL/6 mice. Images obtained via IVIS at different time points (days 1, 3, 7, 13, 17, and 21) show the biodistribution and degradation of PLGA microparticles (n = 3). **b**, Quantification of Cy5 fluorescence signal intensity over time (left) and corresponding area measurements (right) indicating the gradual degradation of PLGA microparticles (n = 3). **c**, Body weight changes of mice over 21 days post iMRAS injection, showing no significant differences, suggesting systemic safety (n = 3). **d**, SEM images of Cy5-labeled PLGA microparticles (left) and histological analysis of the subcutaneous implantation site at day 21 (right). Scale bar: 50 μm. **e**, Biodistribution of iNKT and NK cells in blood on day 5 post iMRAS subcutaneous injection. Percentages of immune cell populations are shown for different treatment groups (Vehicle, iMRAS, iMRAS+αGC) (n = 4). **f**, Immune cell populations in the liver, indicating iNKT and NK cell recruitment in response to iMRAS and iMRAS+αGC treatments (n = 4). **g**, Immune cell populations in the spleen, showing significant increases in iNKT cells following iMRAS+αGC treatment (n = 4). **h**, Blood panel analysis, including white blood cell (WBC), red blood cell (RBC), hemoglobin (HGB), and hematocrit (HCT) levels, at days 1, 15, and 30 post iMRAS injection (n = 4). **i**, Experimental design for evaluating the safety of adoptive cell therapy (ACT) combining subcutaneous human-version iMRAS injection and intravenous human CAR-iNKT cell administration in NSG mice. Tissue collection and terminal analysis were conducted on day 60 (n = 5). **j**, Body weight changes in NSG mice over 60 days following CAR-iNKT + iMRAS treatment (n = 5). **k**, Heatmap of organ damage markers (i.e., urea, ALT, AST, bilirubin, and GLDH) measured in blood samples at days 1, 30, and 60. Data are presented as the mean ± s.e.m. NS, not significant; *P < 0.05, **P < 0.01, ***P < 0.001, ****P < 0.0001, by one-way ANOVA (**e** – **h**) or two-way ANOVA (**j**).

To evaluate the *in vivo* safety and biodistribution of iMRAS, subcutaneous implantation studies were performed in C57BL/6 mice by using Cy5-labeled mouse-version iMRAS microparticles, which were conjugated with mouse CD1d and IL15Tx on their surface. In this study design, iMRAS was tested in two groups: iMRAS+αGC, where αGC was encapsulated within PLGA nanoparticles to enable controlled release and subsequent loading onto CD1d, and iMRAS-αGC, where αGC was absent, resulting in no CD1d-mediated antigen presentation. IVIS imaging over a 21-day period revealed a gradual decline in fluorescence intensity at the implantation site, indicating controlled degradation of the microparticles (Fig. 4a). Analysis of fluorescence signal retention and spatial distribution showed a steady reduction in Cy5 intensity, demonstrating predictable degradation kinetics and minimal off-target dispersion (Fig. 4b). Body weight monitoring showed no significant changes over the 21-day period, demonstrating systemic safety (Fig. 4c). Scanning electron microscope (SEM) images and histological analysis confirmed the structural integrity of the microparticles and the absence of pathological changes in surrounding tissues after 21 days (Fig. 4d). To evaluate the biodistribution and activation kinetics of immune cells following iMRAS treatment, we analyzed their populations in various organs on day 5 post-implantation. Flow cytometry confirmed enhanced recruitment of endogenous iNKT cells across various organs, including the blood, spleen, and liver, in mice treated with iMRAS compared to controls. Additionally, we observed increased recruitment of NK cells in the blood (Fig. 4e), but not in the spleen and liver (Fig. 4f, h), which may be attributed to the effects of IL-15 on NK cell proliferation and circulation. Further analysis of immune cell populations at this time point revealed no significant changes in other leukocyte subsets, including T cells, monocytes, and B cells, indicating that iMRAS selectively enhances iNKT and NK cell responses without broadly altering immune homeostasis (**Supplementary Fig. 6**). Blood analysis confirmed no significant alterations in key hematological parameters, while biochemical profiling of small molecules in circulation further supported systemic safety, indicating localized activation without off-target immune cell accumulation (Fig. 4h). Additionally, hematological and biochemical analysis performed 24 hours post-treatment in C57BL/6 mice further validated iMRAS safety, showing no significant changes in key immune cell populations or metabolic markers (**Supplementary Fig. 7**). Although no significant differences were observed in overall lymphocyte counts, this may be due to iNKT cells constituting only a small fraction (~1%) of total lymphocytes, making their expansion less detectable at a bulk population level. Acute toxicity testing through histological examination of major organs, including the heart, kidney, lung, liver, spleen, and subcutaneous tissue, revealed no significant pathological abnormalities following iMRAS treatment with or without αGC, confirming its biocompatibility (**Supplementary Fig. 8**). Longitudinal immune profiling at days 15 and 45 post-treatment demonstrated that iNKT cells remained effectively activated within the first two weeks, aligning with the drug release kinetics observed in Fig. 1i, whereas by day 45, endogenous immune responses trended toward homeostasis, indicating a return to immune equilibrium (**Supplementary Fig. 9**).

To further validate the safety and functionality of iMRAS in the context of adoptive cell therapy (ACT), we conducted studies in NSG mice using human-version iMRAS. Subcutaneous implantation of iMRAS was performed on day 0, followed by intravenous administration of human CAR-iNKT cells on day 1, with detailed monitoring of systemic and localized effects over a 60-day period (Fig. 4i). Body weight measurements over a 60-day period indicated no adverse effects from the combined treatment (Fig. 4j). Pathological assessment of major organ damage in NSG mice further demonstrated the excellent safety profiles of iMRAS treatment, both with and without αGC implantation (**Supplementary Fig. 10**). Blood panel analyses confirmed the absence of systemic toxicity, with no significant abnormalities in markers of organ function, including urea, alanine aminotransferase (ALT), aspartate aminotransferase (AST), bilirubin, and glutamate dehydrogenase (GLDH) (Fig. 4k). These findings underscore the compatibility of iMRAS with ACT scenarios, showcasing its potential for clinical applications.

### *In vivo* Enhancement of Recruitment and Persistence of Human CAR-iNKT Cells By iMRAS in A Human Solid Tumor Xenograft Mouse Model

**Figure 5:**
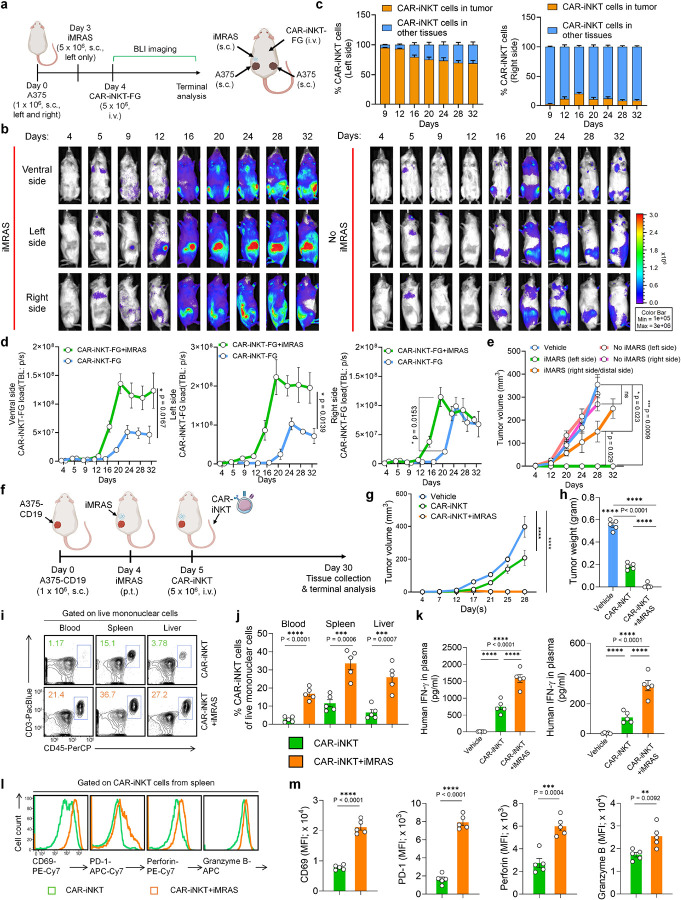
iMRAS effectively recruits human CAR-iNKT cells to solid tumors and activates them to target tumorcells in an A375 human melanoma xenograft mouse model. **a-e**, Studying the recruitment of human CAR-iNKT cells by iMRAS using an A375 human melanoma xenograft mouse model. a, Experimental design. **b**, BLI images showing the presence of CAR-iNKT-FG cells in experimental mice over time. Data from three sides of a single mouse are shown, including the ventral, left, and right sides. **c**, Quantification of the BLI signal proportion in tumor and other tissues (n = 3). **d**, Quantification of the total body BLI signal from the three sides (n = 3). **e**, Tumor volume measurements over time (Vehicle, n = 6; other groups, n = 3). **f-m**, Studying the antitumor capacity of human CAR-iNKT cells with iMRAS using an A375-CD19-FG human melanoma xenograft mouse model. **f**, Experimental design. **g**, Tumor volume measurements over time (n = 5). **h**, Tumor weight measurements at Day 30 (n = 5). i, FACS detection of CAR-iNKT cell percentage in the indicated tissues collected from the experimental mice at Day 30. **j**, Quantification of **i** (n = 5). k, ELISA analyses of the human cytokine levels in mouse plasma collected at Day 30. **l**, FACS analyses of the expressions of effector molecule (i.e., CD69) and cytotoxic molecules (i.e., Perforin and Granzyme B) of CAR-iNKT cells in mouse spleen. **m**, Quantification of **l** (n = 5). Data are presented as the mean ± s.e.m. NS, not significant; *P < 0.05, **P < 0.01, ***P < 0.001, ****P < 0.0001, by Student’s t test **(j** and **m**), one-way ANOVA (**h** and **k**), or two-way ANOVA (**d**, **e**, and **g**).

To investigate CAR-iNKT cell recruitment and activation by iMRAS *in vivo*, we established a human solid tumor xenograft model (Fig. 5a). In this model, A375 cells were utilized as tumor cells without CD19 overexpression, thereby allowing us to examine TCR/antigen-induced iNKT recruitment mediated by iMRAS and reducing any bias from CAR recognition. The CAR-iNKT cells were labeled with FG (denoted as CAR-NKT-FG cells), enabling real-time monitoring of the pharmacokinetics and pharmacodynamics (PK/PD) of these cells *in vivo* under varying conditions. To set up the *in vivo* model, 1 × 10^6^ A375 tumor cells were subcutaneously injected into both the left and right flanks of NSG mice. After three days, iMRAS was implanted into the left flank only. Subsequently, CAR-iNKT-FG cells were administered via intravenous injection, and bioluminescence imaging (BLI) was employed to monitor the PK/PD of the CAR-iNKT-FG cells *in vivo* (Fig. 5a).

Following intravenous injection into NSG mice, CAR-iNKT-FG cells exhibited efficient expansion, peaking approximately 2–3 weeks post-injection before stabilizing (Fig. 5b). Upon implantation of iMRAS in the left flank, CAR-iNKT-FG cells were rapidly recruited to this site within five days, accumulating near the tumor and undergoing rapid expansion (Fig. 5b, c). In contrast, the tumor on the right flank exhibited significantly fewer accumulated CAR-iNKT-FG cells, with the majority of signals emanating from other tissues such as the spinal cord, femur, and lung (Fig. 5b, c).

In comparison to tumor-bearing mice without iMRAS implantation, we observed some expansion of CAR-iNKT-FG cells; however, the levels were markedly lower than those observed in mice with iMRAS implantation, across all measured sites: ventral side, left flank, and right flank (Fig. 5b, d). In the absence of iMRAS, most CAR-iNKT-FG cells localized to other tissues, such as the lung and bone, with limited tumor homing attributed to the lack of TCR signaling on the tumor surface, resulting in inadequate recruitment and activation of CAR-iNKT-FG cells (Fig. 5b, d). Importantly, the total body signal of CAR-iNKT-FG cells was also lower in mice without iMRAS when compared to those with iMRAS (Fig. 5b, d). This indicates that iMRAS not only facilitates the recruitment of CAR-iNKT cells to solid tumor sites but also systemically activates them, enhancing their expansion and functionality. This improved performance is likely a result of both TCR stimulation and IL-15 support provided by the iMRAS platform^21,22,41^.

In alignment with the recruitment and activation of CAR-iNKT-FG cells by iMRAS, these cells demonstrated effective tumor elimination on the left side of the experimental mice, where iMRAS was implanted (Fig. 5e). Additionally, the tumors on the right side, representing distal tumor sites, were also significantly suppressed, which is attributed to the systemic activation of CAR-iNKT-FG cells by iMRAS (Fig. 5e).

### *In vivo* Enhancement of Antitumor Capacity and Activation of Human CAR-iNKT Cells by iMRAS in a Solid Tumor Xenograft Model

We evaluated the antitumor capacity and functionality of CAR-iNKT cells enhanced by iMRAS using an A375-CD19 human melanoma xenograft mouse model. A375-CD19 tumor cells were subcutaneously injected into mice, and iMRAS was implanted on the tumor side. Subsequently, CD19-targeting CAR-iNKT cells were administered via intravenous injection. Tumor size was monitored over time, and tumor weight was measured on Day 30 at the study’s conclusion (Fig. 5f).

Interestingly, while CAR-iNKT cells demonstrated some antitumor capacity in the absence of iMRAS, they were limited in their ability to control tumor growth (Fig. 5g, h). This inefficacy is likely attributed to inefficient recruitment and infiltration of CAR-iNKT cells, a common phenomenon observed in CAR-engineered cell therapies targeting solid tumors^42–44^. However, with the assistance of iMRAS, we observed significant tumor suppression and, in some cases, complete tumor elimination by CAR-iNKT cells (Fig. 5g, h).

Due to the small size of the tumors, we were unable to extract CAR-iNKT cells directly from the tumor sites. Instead, we analyzed the phenotype and functionality of CAR-iNKT cells in other tissues, including blood, spleen, and liver. Notably, following iMRAS implantation, we observed an enrichment of CAR-iNKT cells across all examined tissues, indicating enhanced overall activation and improved persistence of these cells *in vivo* (Fig. 5i, j). Corresponding to the increased numbers of CAR-iNKT cells, we detected elevated levels of effector molecules, including human IFN-γ and TNF-α, in the mouse plasma (Fig. 5k). Furthermore, after iMRAS activation, CAR-iNKT cells significantly upregulated effector markers such as CD69 and produced increased levels of cytotoxic molecules, including Perforin and Granzyme B (Fig. 5l, m). This enhancement in *in vivo* proliferation and activation resulted in superior solid tumor cytotoxicity by CAR-iNKT cells.

### *In vivo* Systemic Enhancement of Antitumor Capacity and Activation of Human CAR-iNKT Cells by iMRAS in A Blood Cancer Xenograft Model

**Figure 6:**
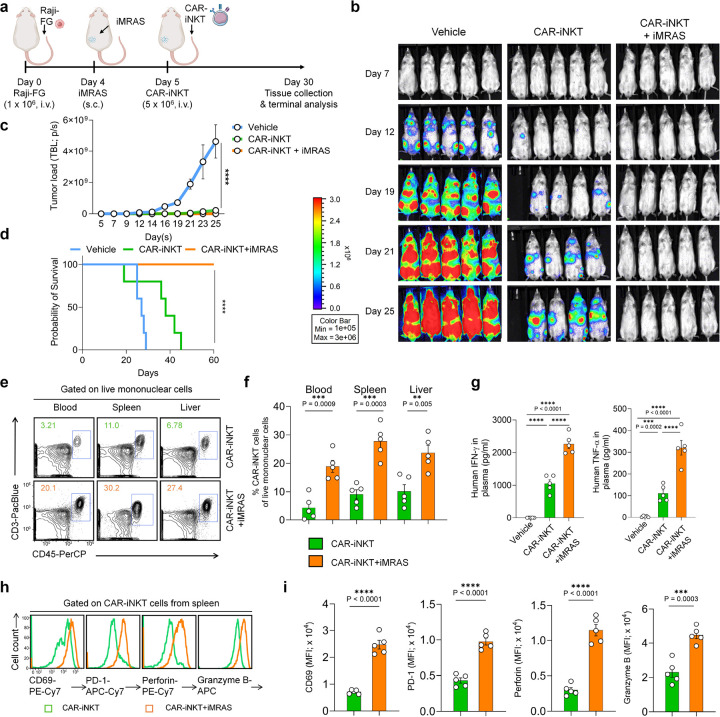
iMRAS systemically activates CAR-iNKT cells to eliminate blood cancer in a Raji human lymphoma xenograft mouse model. **a**, Experimental design. **b**, BLI images showing the presence of tumor cells in experimental mice over time. **c**, Quantification of **b** (n = 5). **d**, Kaplan-Meier survival curves of experimental mice over time (n = 5). **e**, FACS detection of human CAR-iNKT cells in the blood, spleen, and liver collected from the experimental mice on day 30. **f**, quantification of **e** (n = 5). **g**, ELISA analysis of human IFN-γ and TNF-α in plasma samples collected from the experimental mice on day 30. **h**, FACS measurements of the surface expression of CD69 and PD-1, as well as intracellular expression of Perforin and Granzyme B in CAR-iNKT cells isolated from the mouse spleen. **i**, quantification of **h** (n = 5). Data are presented as the mean ± s.e.m. NS, not significant; *P < 0.05, **P < 0.01, ***P < 0.001, ****P < 0.0001, by Student’s t test (**f** and **i**) or one-way ANOVA (**c** and **g**).

Given that iMRAS can recruit both iNKT and CAR-iNKT cells while also systemically activating iNKT cells, leading to significant enhancements in their persistence and expansion *in vivo* (Fig. 5b–e), we investigated the systemic functionality of CAR-iNKT cells using a Raji-FG human lymphoma xenograft mouse model (Fig. 6a). 1 × 10^6^ Raji-FG cells were administered via intravenous injection into NSG mice, followed by subcutaneous implantation of iMRAS. Subsequently, human CAR-iNKT cells were also administered intravenously, and BLI was utilized to monitor tumor growth (Fig. 6a).

CAR-iNKT cells alone were effective in suppressing tumor cell growth and prolonging the survival of the experimental mice (Fig. 6b–d). However, with the assistance of iMRAS, we observed complete elimination of Raji tumor cells *in vivo*, with no observed mortality in the mice (Fig. 6b–d). The significant enhancement in antitumor capacity was associated with notable expansion of CAR-iNKT cells across multiple organs, including the blood, spleen, and liver (Fig. 6e, f). This expansion correlated with increased effector functions of CAR-iNKT cells, as evidenced by elevated levels of effector cytokines in plasma, along with enhanced expression of effector markers and production of cytotoxic molecules (Fig. 6g–i).

Overall, these findings from *in vivo* humanized mouse models indicate that iMRAS significantly enhances CAR-iNKT cell efficacy through two primary mechanisms: First, for solid tumors, iMRAS effectively recruits CAR-iNKT cells to tumor sites, thereby bolstering their tumor-killing capacity. Second, it systemically activates CAR-iNKT cells, improving their effector function and promoting *in vivo* expansion, which ultimately leads to more functional CAR-iNKT cells capable of targeting metastatic tumor cells, a leading cause of mortality in many solid tumors^45^.

### Superior Antitumor Efficacy of CAR-iNKT Cells with iMRAS Compared to Conventional CAR-T cell in Blood Cancer and Solid Tumor Xenograft Models

We then compared the *in vivo* antitumor capacity of CAR-iNKT cells with iMRAS to that of conventional CAR-T cells, which are the frontline cell-based therapy for many cancers, particularly hematological malignancies^46–48^. To conduct this evaluation, we utilized xenograft mouse models of both A375-CD19 human melanoma and Raji-FG human lymphoma (**Supplementary Fig. 11**).

Notably, in both solid tumor and blood cancer models, conventional CD19-targeting CAR-T cells were unable to fully eliminate tumor cells, leading to tumor relapse and ultimately, mouse mortality (Supplementary Fig. 11). The antitumor efficacy of CAR-T cells was comparable to that of CAR-iNKT cells alone, but significantly lower than that of CAR-iNKT cells combined with iMRAS implantation (**Supplementary Fig. 11**). These findings suggest that our iMRAS platform substantially enhances the efficacy of CAR-iNKT cell-based therapy, surpassing the performance of conventional CAR-T cell therapy in cancer treatment. Importantly, only CAR-iNKT cells benefit from iMRAS due to their invariant TCR and unique recognition of lipid antigens. In contrast, CAR-T cells possess a highly diverse TCR repertoire that recognizes a wide range of peptide antigens, lacking a uniform and efficient system for recruitment and activation based on TCR recognition.

## Discussion

iMRAS replicates the functionality of natural immune signals by integrating finely tuned mechanical properties with optimized cytokine release and ligand presentation, providing both activation signals and chemotactic signals to enhance CAR-iNKT cell recruitment and stimulation, thereby establishing a highly immunostimulatory microenvironment. By incorporating CD1d presenting αGC and IL-15Tx complexes, iMRAS enhances the presentation of signaling molecules, prolonging ligand half-life and delivering more potent and sustained stimulation to iNKT cells, thereby promoting robust activation and expansion. Recent studies have demonstrated that immune cells are highly responsive to mechanical cues within the microenvironment, further supporting the rationale for iMRAS’s biomimetic design to enhance CAR-iNKT cell functionality^49–52^. However, research specifically investigating the impact of mechanical cues on iNKT cells remains limited, highlighting the novelty of our approach in leveraging biomechanical properties, together with biochemical signals, to optimize CAR-iNKT activation and expansion. This advancement represents a crucial step toward developing more effective and durable cancer immunotherapies with CAR-iNKT cells.

The inclusion of αGC-loaded PLGA nanoparticles within iMRAS enables a controlled and sustained release of αGC, initially providing chemotactic signals to attract iNKT cells to the vicinity of iMRAS. This localized recruitment ensures that iNKT cells are positioned near iMRAS for subsequent activation. Since iMRAS is implanted near the tumor, this strategy effectively concentrates immune activation within the TME, overcoming the limitations of transient stimulation in existing therapies and supporting long-term anti-tumor efficacy. This concept aligns with the *in vivo* ‘charging station’ model, where iMRAS serves as a focal point for sustained immune engagement. Nanomedicine has emerged as a pivotal tool in cancer therapy due to its ability to improve drug retention and targeted delivery. One of the major challenges in nanomedicine is rapid clearance of nanoparticles *in vivo*, which limits their therapeutic efficacy^53–55^. By integrating PLGA nanoparticles within the iMRAS scaffold, we effectively circumvent this limitation, ensuring prolonged local retention of nanoparticles and a sustained therapeutic effect for up to 15 days. This approach demonstrates the potential of combining biomaterial-based scaffolds with nanomedicine to enhance therapeutic outcomes in cancer immunotherapy. Ensuring the safety of the iMRAS system is a vital aspect of its clinical application. The iMRAS microspheres and the key components, such as alginate, PLGA, antibodies, and CD1d tetramers, are FDA-approved for use in medical devices and can be used for clinical applications^56–58^.

One of the major challenges in solid tumor therapy is addressing metastasis, which remains a significant hurdle in achieving long-term disease control^59–61^. In addition to local effects, iMRAS has the capacity to activate iNKT cells that may circulate throughout the body and activate other endogenous immune cells, achieving a systemic immune response and cancer therapy at distant sites. This strategy enhances the overall therapeutic impact and broadens the applicability of CAR-iNKT therapies to metastatic and multi-focal tumors. Our concept aligns with the systemic circulation framework illustrated in Fig. 1a, where iMRAS-activated iNKT cells enter circulation and contribute to immune surveillance and tumor eradication beyond the primary tumor site. Notably, CAR-iNKT cells stimulated by iMRAS can infiltrate distant metastatic tumors, amplifying their cytotoxic effects beyond the local microenvironment. This systemic immune modulation further supports the potential of iMRAS as an advanced platform for enhancing CAR-iNKT therapies against disseminated malignancies.

CAR-iNKT cells have demonstrated robust antitumor efficacy in preclinical and clinical studies, offering a unique advantage through their ability to leverage multiple tumor-targeting mechanisms^12,15,22^. Strategies such as cytokine engineering, including the overexpression of IL-12 and IL-15, have been utilized to improve the *in vivo* persistence and cytotoxicity of CAR-iNKT cells^20,62^. However, challenges remain in overcoming tumor microenvironment barriers, particularly in solid tumors^63,64^. Our study highlights the potential of iMRAS to address this challenge: by mimicking antigen presentation and providing sustained cytokine stimulation, iMRAS efficiently recruits CAR-iNKT cells to tumor sites and systemically activates them, enhancing their expansion and functionality. These findings position the iMRAS system as a promising tool to optimize CAR-iNKT cell-based therapies.

The iMRAS system, designed with a biomimetic structure that functionalizes its surface with CD1d molecules presenting αGC and IL-15Tx complexes to mimic APCs, represents an innovative first-generation platform with significant potential. Its flexibility allows for various modifications to enhance its function and broaden its applications. Future versions could incorporate additional cytokines, chemokines, or immune checkpoint inhibitors to further amplify its ability to recruit and activate immune cells. For instance, integrating IL-21 into the iMRAS system could specifically increase the frequency of CD62L-positive memory-like CAR-iNKT cells, thereby improving long-term disease-free survival^24^.

Similarly, chemokines such as CCL3, CXCL16, and CX3CL1 could be incorporated to enhance iNKT cell trafficking, ensuring more efficient activation at the iMRAS site^65–68^. Immune checkpoint inhibitors such as anti-LAG-3 or anti-PD-1 antibodies could also be included to further boost CAR-iNKT cell activation and proliferation, significantly increasing their IFN-γ secretion and therapeutic efficacy^69^.

Importantly, the biocompatible materials used to construct iMRAS provide a safe foundation for further innovation. This safety profile supports their application in diverse anatomical sites, opening opportunities for broader cancer therapy potential. Injectable hydrogels, for example, have been extensively explored as minimally invasive, tissue-compatible platforms for treating glioblastoma (GBM) and hepatocellular carcinoma (HCC) by serving as systems for effective drug delivery or platforms for capturing and eradicating residual tumor cells^70–74^. Utilizing such approaches, iMRAS could also be injected *in situ* to target GBM, HCC, lung cancer^75^, and other challenging disease models, when combined with CAR-iNKT cell-based immunotherapies.

## Methods

### Mice

NOD.Cg-Prkdc^SCID^Il2rg^tm1Wjl^/SzJ (NOD/SCID/IL-2Rγ^−/−^, NSG) mice and C57BL/6J (B6), B6.SJL-Ptprc^a^Pepc^b^/BoyJ (CD45.1) mice were maintained in the animal facilities at the University of California, Los Angeles (UCLA). Male or female mice aged 6–10 weeks were used for all experiments unless otherwise specified. Sex was not considered in the study design and analysis, as no significant differences were observed in the models. All animal experiments were approved by the Institutional Animal Care and Use Committee of UCLA. All mice were bred and maintained under specific pathogen-free conditions, and all experiments were conducted in accordance with the animal care and use regulations of the Division of Laboratory Animal Medicine at the UCLA. For tumor studies, the experimental mice were randomly assigned to treatment groups to avoid statistically significant differences in the baseline tumor burden.

### Media and reagents

α-Galactosylceramide (αGC, KRN7000, cat. no. 867000) was purchased from Avanti Polar Lipids. Recombinant human IL-2 (cat. no. 200-02), IL-7 (cat. no. 200-07), and IL-15 (cat. no. 200-15) were purchased from Peprotech. Fetal bovine serum (FBS, lot no. 2087050), and beta-mercaptoethanol (β-ME, cat. no. 1610710) were purchased from Sigma. Penicillin-streptomycin-glutamine (PSG, cat. no. 10-378-016), MEM non-essential amino acids (cat. no. 11-140-050), HEPES buffer solution (cat. no. 15630080), and sodium pyruvate (cat. no. 11360070) were purchased from Gibco. Normocin was purchased from Invivogen (cat. no. ant-nr-2).

Homemade C10 medium consisted of RPMI 1640 cell culture medium (cat. no. MT10040CV) supplemented with (10% vol/vol) FBS, (1% vol/vol) PSG, (1% vol/vol) MEM non-essential amino acids, 10 mM HEPES, 1 mM sodium pyruvate, 50 μM β-ME, and 100 μg/ml Normocin. Homemade D10 medium consisted of DMEM medium (cat. no. MT10013CV) supplemented with (10% vol/vol) FBS, (1% vol/vol) PSG, and 100 μg/mL Normocin. Homemade R10 medium consisted of RPMI supplemented with (10% vol/vol) FBS, 1% (vol/vol) PSG, and 100 μg/ml Normocin.

Poly Lactic-co-Glycolic Acid (PLGA), 50:50 was purchased from Polysciences (cat. no. 23987). PRONOVA^®^ UP VLVG (cat. no. 42000501-5G), Sodium carboxymethyl cellulose (cat. no. 419338), Ethylenediaminetetraacetic acid calcium disodium salt hydrate (cat. no. 340073), Calcium chloride (cat. no. C4901) and Acetic acid (cat. no. 338826) were purchased from sigma. Biotinylated Human IL-15 R alpha (cat. no. ILA-H82F4) and human IL-15 (cat. no. IL5-H4117) were purchased from ACROBiosystems.

### Fabrication of αGC loaded PLGA nanoparticles for iNKT cell recruitment

PLGA nanoparticles were fabricated using the emulsion-solvent evaporation technique. First, αGC and PLGA were dissolved together in dichloromethane to form the organic phase. Simultaneously, a 1% poly(vinyl alcohol) (PVA) solution was prepared as the aqueous phase. Next, the organic phase (containing αGC and PLGA) was added dropwise into the PVA solution under ultrasonication for 3 minutes, forming a stable emulsion. Following emulsification, the organic solvent was removed by continuous stirring at room temperature in 0.1% PVA solution overnight, allowing the PLGA to precipitate and encapsulate αGC within the forming nanoparticles. The resulting nanoparticles were collected by centrifugation, washed extensively to remove any residual solvent or stabilizer, and lyophilized for long-term storage.

### PLGA drug loading and releasing profile

To evaluate drug loading efficiency, we used dansylated αGC as a fluorescent tracer. Dichloromethane was added to dissolve the PLGA nanoparticles, and the mixture was vortexed or gently sonicated until fully solubilized. To quantify αGC content, we pipetted 100 μL of each sample extract into a 96-well plate and measured fluorescence using a plate reader at an excitation wavelength of 335 nm and an emission wavelength of 518 nm. Absorbance values were compared against a standard curve generated from αGC standards to calculate the total αGC recovered. The loading efficiency (%) was determined by dividing the measured αGC mass (adjusted for sample dilution) by the theoretical αGC mass originally incorporated into the nanoparticles.

For in vitro release kinetics, PLGA–αGC nanoparticles were suspended in DMEM and incubated at 37°C with 5% CO_2_ for 30 days to simulate physiological conditions. At predetermined time points, aliquots of the nanoparticle suspension were withdrawn and centrifuged to separate the supernatant (containing released αGC) from the nanoparticle pellet. The collected supernatants were lyophilized and stored at −20°C until analysis, while an equivalent volume of fresh DMEM was added back to the nanoparticle pellet to maintain sink conditions throughout the experiment.

### iMRAS manufacturing

We designed the microfluidic device using AutoCAD 2022 and fabricated it via a standard photolithography process. To create the master mold, we spin-coated a 4-inch silicon wafer with photosensitive epoxy (SU-8 2015, MicroChem) to a thickness of 13 μm, followed by a 95°C bake for 6 minutes to harden the coating. Ultraviolet light was then applied through a chrome mask to transfer the channel patterns. After exposure, the wafer was developed in SU-8 developer for 10 minutes, rinsed with isopropyl alcohol, and dried. For microfluidic chip fabrication, we prepared a PDMS mixture by combining the pre-polymer and curing agent, then poured it onto the master mold and cured it at 65°C for 3 hours. Once cured, the PDMS slab was punched to form inlets and outlets, followed by channel cleaning with 50% ethanol. To facilitate bonding, the slab underwent oxygen plasma treatment (Plasma Prep II, SPI Supplies) and was then heated at 65°C for 30 minutes to strengthen the attachment. The bonded device was left at room temperature for 24 hours to enhance hydrophobicity. To optimize iMRAS production, we designed a 3D-printed reservoir using an Elegoo 4K printer, which was sterilized with 70% ethanol before attachment to the microfluidic device. This reservoir was capable of holding up to 5 mL of iMRAS microparticles produced in the oil phase, enabling efficient manual collection. Throughout the production process, we monitored alginate bead size and production rate under a microscope to ensure consistency and quality control.

iMRAS were produced in the microfluidic device using a pH-induced internal gelation method. The core stream consisted of a solution containing 3% alginate (very low viscosity grade, molecular weight 70 kDa, NovaMatrix), 1% CMC (to stabilize laminar flow), Ca–EDTA and 2mg PLGA nanoparticles mixed in deionized water. The sheath stream included 1% surfactant 157 FSH and 0.5% acetic acid, dissolved in Novec 7500. Both solutions were sterilized with a 0.22 μm filter before use. The core and sheath fluids were introduced into the microfluidic device via separate inlets, driven by syringe pumps (Harvard Apparatus). Microbeads were formed within the device and temporarily stored in a reservoir before being collected into a tube containing 20% perfluoro-1-octanol (Sigma) and 0.2% acetic acid in fluorocarbon oil. This collection solution promoted particles crosslinking. Next, HEPES-C buffer (20 mM HEPES, 140 mM sodium chloride, 5 mM potassium chloride, 2 mM calcium chloride, pH 7.2) was added to the collection tube. The tube was centrifuged at 1,000 g for 1 minute to transfer the microparticles from the oil phase to the buffer. The collected microparticles were then rinsed twice with buffer at 6,000 g for 5 minutes to ensure thorough cleaning. Finally, the clean microparticles were stored at 4 °C for further experiments.

### Preparation of iMRAS for iNKT cell stimulation

iMRAS with precise ligand organization can improve CAR-iNKT cell activation. This enhancement leads to improved expansion, persistence, and therapeutic efficacy. iMRAS were prepared using tetrazine–TCO ligation to click proteins and cytokine to microparticle. In short, 4 million microparticles were resuspending in MES buffer containing 40mg/ml 1-ethyl-3-(3-dimethylaminopropyl) carbodiimide hydrochloride and 30 mg/ml sulfo-NHS, then incubated for 50 mins to activate the carboxylic groups. Afterward, the particles were washed with HEPES-CT buffer (HEPES with 2 mM calcium chloride and 0.5% Tween-20) to adjust the pH to 7.2. TCO−PEG6-amine was added at 2 μmol per million particles. This mixture reacted overnight at room temperature with gentle rolling to ensure uniform mixing. Next, the particles were then dialyzed using a 1000 kDa dialysis bag to remove excess TCO. Proteins were conjugated with tetrazine following the manufacturer’s instructions. For CAR-iNKT cell activation, CD1d and IL-15Ra (mixed 1:1) were used. The antibody/protein mixture was concentrated to 100 μl in PBS using an Amicon Ultra-2 spin column. Tetrazine-PEG5-NHS was added at a 1:5 molar ratio. The reaction proceeded for 30 minutes. The resulting antibody/protein–tetrazine complex was desalted using a spin column and washed five times with PBS to remove unreacted reagents. This purified complex was mixed with glycerol (1:1 ratio) and stored at −20 °C. During the conjugation process, human CAR-iNKT cell-specific iMRAS utilized human CD1d, whereas mouse-specific iMRAS utilized mouse CD1d.

### *In vivo* degradation tracking

To assess the *in vivo* degradation profile of PLGA nanoparticles, a Cy5-conjugated PLGA polymer (CD Bioparticles, CDV034) was synthesized and used to fabricate the nanoparticles. The fluorescently labeled iMRAS were then administered subcutaneously into animal models, and their biodistribution and degradation were monitored by near-infrared fluorescence imaging at predetermined time points.

### AFM for IMRAS mechanical property and cell mechanics methods

On the day of the experiment, APCs (primary monocytes) were isolated from human PBMCs using the CD14 MicroBeads kit (Miltenyi Biotec; cat. no. 130-050-201). iMRAS and APCs were then mounted on a JPK NanoWizard 4a BioScience AFM. A Bruker SAA-SPH-1UM probe, with a spring constant (k) of approximately 0.25 N/m, was employed for indentation. The exact k value for each probe was determined by laser Doppler velocimetry calibration and applied to the respective measurements. After collecting force spectroscopy data, the resulting force curves were fitted to a Hertz/Sneddon model using JPK Data Processing (v.3.4) to determine Young’s modulus. To measure viscoelasticity, the height was held constant at 10 nN on the surfaces of iMRAS and APCs. The stress relaxation profile was then obtained by recording the vertical deflection force over the relaxation period.

### SEM imaging of iMRAS and EDS scanning

We prepared lyophilized iMRAS samples by mounting them on suitable stubs and, when necessary, sputter-coated them with a thin layer of gold to enhance conductivity. The samples were then placed in the SEM chamber, where we adjusted the working distance and set the accelerating voltage to 10 kV. Once optimal imaging conditions were established, we captured high-resolution micrographs. For energy-dispersive X-ray spectroscopy (EDS) analysis, we focused the electron beam on specific regions of interest and performed elemental mapping across the entire sample. Spectral data were collected and processed using EDS software to identify and quantify the elemental composition.

### Lentiviral vectors

Lentiviral vectors used in this study were all constructed from the parental lentivector pMNDW^76,77^. The Lenti/CAR19 vector was generated by inserting a synthetic gene encoding a CD19-targeting CAR into pMNDW. The Lenti/FG vector was generated by inserting a synthetic gene encoding firefly luciferase and enhanced green fluorescent protein (EGFP) dual reporter (FG) gene into the lentiviral backbone. The Lenti/CD19 vector was generated by inserting a synthetic gene encoding human CD19 into pMNDW. The synthetic gene fragments were obtained from GenScript and Integrated DNA Technologies. Lentiviruses were produced using human embryonic kidney 293T cells purchased from ATCC, following a standard transfection protocol with Trans-IT-Lenti Transfection Reagent (Mirus Bio, SKU MIR 6604) and a concentration protocol^11,17^ using Amicon Ultra Centrifugal Filter Units (MilliporeSigma, cat. no. UFC9010), according to the manufacturer’s instructions.

### Cell lines

Human melanoma cell line A375 (cat. no. CRL-1619), Burkitt’s lymphoma cell line Raji (cat. no. CCL-86), and human embryonic kidney (HEK) 293T (cat. no. CRL-3216) were purchased from the ATCC. To generate stable tumor cell lines overexpressing human CD19 and/or FG dual reporters, parental tumor cell lines were transduced with lentiviral vectors encoding the desired gene(s). 72 hours after lentivector transduction, cells were sorted via flow cytometry to isolate gene-engineered cells for stable line generation. Five stable tumor cell lines were utilized in this study, including A375, A375-FG, A375-CD19, A375-CD19-FG, and Raji-FG cells.

### Antibodies and flow cytometry

Fluorochrome-conjugated antibodies specific for human CD45 (Clone HI30, cat. no. 982318, PerCP-conjugated, 1:500 dilution), TCRαβ (Clone IP26, cat. no. 306716, PacBlue-conjugated, 1:25 dilution), CD3 (Clone HIT3a, cat. no. 300329, PacBlue-conjugated, 1:500 dilution), CD4 (Clone OKT4, cat. no. 317408, FITC-conjugated, 1:400 dilution), CD8 (Clone SK1, cat. no. 344714, APC-Cy7-conjugated, 1:500 dilution), CD69 (Clone FN50, cat. no. 310912, PE-Cy7-conjugated, 1:50 dilution), CD1d (Clone 51.1, cat. no. 350310, PE-Cy7-conjugated, 1:50 dilution), PD-1 (Clone EH12.2H7, cat. no. 329922, APC-Cy7-conjugated, 1:50 dilution), Granzyme B (Clone QA16A02, cat. no. 372204, APC-conjugated, 1:4,000 dilution), and Perforin (Clone dG9, cat. no. 308126, PE-Cy7-conjugated, 1:50 dilution) were purchased from BioLegend. Fluorochrome-conjugated antibodies specific for mouse CD45 (Clone 30-F11, cat. no. 103130, PerCP-conjugated, 1:200 dilution), CD3 (Clone 17A2, cat. no. 100236, APC-conjugated, 1:200 dilution), CD19 (Clone 1D3/CD19, cat. no. 152412, APC-Cy7-conjugated, 1:200 dilution), CD4 (Clone GK1.5, cat. no. 100406, FITC-conjugated, 1:200 dilution), CD8 (Clone 53-6.7, cat. no. 100708, PE-conjugated, 1:200 dilution), CD11b (Clone M1/70, cat. no. 101216, PE-Cy7-conjugated, 1:200 dilution), CD14 (Clone M14-23, cat. no. 150106, PE-conjugated, 1:200 dilution), and NK-1.1 (Clone S17016D, cat. no. 156508, FITC-conjugated, 1:200 dilution) were purchased from BioLegend. Fluorochrome-conjugated antibodies specific for human iNKT TCR Vα24-Jβ18 (Clone 6B11, PE-conjugated, 1:20, cat. no. 552825) were purchased from BD Biosciences. A goat anti-mouse IgG F(ab’)2 secondary antibody was purchased from ThermoFisher (cat. no. A-11001) APC Streptavidin (cat. no. 405207, 1:1000 dilution) was purchased from BioLegend. Fixable ViabilityDye eFluor506 (e506, 1:500, cat. no. 65-0866-14) was purchased from Affymetrix eBioscience. Mouse Fc Block (anti-mouse CD16/32, cat. no. 553141) was purchased from BD Biosciences. Human Fc Receptor Blocking Solution (TrueStain FcX, cat. no. 422302) was purchased from BioLegend. In our study, note the use of antibodies with identical clones but differing conjugated fluorochromes, with one typical antibody listed herein.

All flow cytometry staining was performed following standard protocols, along with specific instructions provided by the manufacturer of each antibody. Stained cells were analyzed using a MACSQuant Analyzer 10 flow cytometer (Miltenyi Biotech), adhering to the manufacturer’s guidelines. Data analysis was conducted using FlowJo software v.9 (BD Biosciences).

### Immunofluorescence staining and microscopy

We first fixed the samples in 4% paraformaldehyde (PFA) in phosphate-buffered saline (PBS) for 15 min at room temperature, followed by permeabilization with 0.2% Triton X-100 in PBS for 10 min. To prevent non-specific binding, we then incubated the samples in 5% normal donkey serum in PBS for 30 min at room temperature. After blocking, we applied primary antibodies diluted in blocking solution and incubated the samples overnight at 4°C. The next day, we performed three PBS washes before incubating the samples with secondary antibodies for 1 h at room temperature in the dark. To visualize nuclei, we counterstained the samples with 4′,6-diamidino-2-phenylindole (DAPI) for 5 min. Finally, we mounted the coverslips using an anti-fade mounting medium, sealed them onto glass slides, and captured images using a fluorescence and confocal microscope while maintaining consistent exposure settings across all samples.

### Enzyme-linked immunosorbent cytokine assays (ELISAs)

Mouse sera or culture supernatants were collected for cytokine ELISA analysis following a standard protocol from BD Biosciences. The coating and biotinylated antibodies for detecting human IFN-γ, TNF-α, and IL-2 (coating antibodies, cat. no. 551220, cat. no. 551221, and cat. no. 554563, respectively; biotinylated detection antibodies, cat. no. 554511, cat. no. 554550, and cat. no. 555040, respectively) were purchased from BD Biosciences. The streptavidin–horseradish peroxidase (HRP) conjugate (cat. no. 18410051) was obtained from Invitrogen. Human IFN-γ and TNF-α standards (cat. no. 29-8319-65 and no. 29-8329-65, respectively) were sourced from eBioscience. The 3,3′,5,5′-tetramethylbenzidine (TMB; cat. no. 51200048) substrate was purchased from Kirkegaard & Perry Laboratories (KPL, Gaithersburg, MD, USA). Absorbance at 450 nm was measured using an Infinite M1000 microplate reader (Tecan, Morrisville, NC, USA).

### Generation of human iNKT and CAR-redirected iNKT (CAR-iNKT) cells

Human iNKT and CAR-iNKT cells were generated from healthy donor-derived peripheral blood mononuclear cells (PBMCs), which were provided by the UCLA/CFAR Virology Core Laboratory without identifying information, in compliance with federal and state regulations.

Healthy donor PBMCs were MACS-sorted using Anti-iNKT Microbead (Miltenyi Biotech; cat. no. 130-094-842) labeling to enrich human iNKT cells. A starting quantity of 5 × 10^8^ PBMCs is typically used to isolate iNKT cells. Donor-matched PBMCs were loaded with αGC (by culturing 1 × 10^7^ PBMCs in 5 ml C10 medium containing 5 mg/ml αGC for 1 hour), irradiated at 6,000 rads, then used to stimulate iNKT cells (denoted as αGC/PBMCs). The sorted iNKT cells were then stimulated with donor-matched irradiated αGC/PBMCs at a ratio of 1:1 and cultured in C10 medium supplemented with human IL-7 (10 ng/mL) and IL-15 (10 ng/mL) for 2–3 weeks. To generate iNKT-FG cells, Lenti/FG lentivector was used to transduce iNKT cells 2 days post αGC/PBMC stimulation. At 72 hours post-lentivector transduction, FG expression in engineered iNKT cells was confirmed by flow cytometry, identifying GFP^+^ cells. The resulting iNKT-FG cells were then further purified using FACS. To generate CD19-targeting CAR-iNKT cells, Lenti/CAR19 lentivector was used to transduce iNKT cells 2 days post αGC/PBMC stimulation. At 72 hours post-lentivector transduction, CAR expression in engineered iNKT cells was confirmed by flow cytometry, identifying CAR^+^ cells. The resulting CAR-iNKT cells were then further purified using FACS. To generate CAR-iNKT-FG cells, Lenti/FG and Lenti/CAR19 lentivectors were used to transduce iNKT cells 2 days post αGC/PBMC stimulation. At 72 hours post-lentivector transduction, the expression levels of CAR and FG were confirmed by flow cytometry, identifying CAR^+^GFP^+^ cells. The resulting CAR-iNKT-FG cells were then further purified using FACS.

### Generation of human CAR-redirected conventional T (CAR-T) cells

Non-treated tissue culture 24-well plates (Corning; cat. no. 3738) were coated with Ultra-LEAF Purified Anti-Human CD3 Antibody (Clone OKT3; BioLegend; cat. no. 317326) at 1 μg/ml (500 μl per well), at room temperature for 2 h or at 4 °C overnight. Healthy donor PBMCs were resuspended in the C10 medium supplemented with 1 μg/ml Ultra-LEAF Purified Anti-Human CD28 Antibody (Clone CD28.2, BioLegend; cat. no. 302934) and 30 ng/ml IL-2, followed by seeding in the precoated plates at 1 × 10^6^ cells per ml (1 ml per well). On day 2, cells were transduced with Lenti/CAR19 lentiviruses for 24 hours. The resulting CD19-targeting CAR-T cells were expanded for about 2 weeks in C10 medium and cryopreserved for future use.

### Single cell RNA sequencing (scRNA-seq)

Human CAR-iNKT cell samples were sorted (identified as iNKT TCR^+^CD3^+^ cells) by using a flow cytometer and delivered to the UCLA TCGB Core for library construction and scRNA-seq. Cells were quantified by using a Cell Countess II automated cell counter (Invitrogen/Thermo Fisher Scientific). A total of 10,000 cells from each experimental group were loaded on the Chromium platform (10X Genomics), and libraries were constructed using the Chromium Next GEM Single Cell 3’ Kit v3.1 and the Chromium Next GEM Chip G Single Cell Kit (10X Genomics), according to the manufacturer’s instructions. Library quality was assessed using the D1000 ScreenTape on a 4200 TapeStation System (Agilent Technologies). Libraries were sequenced on an Illumina NovaSeq using the NovaSeq S4 Reagent Kit (100 cycles; Illumina).

For cell clustering and annotation, the merged digital expression matrix generated by Cellranger was analyzed using an R package Seurat (v.4.0.0) following the official website guidelines. Briefly, after filtering the low-quality cells, the expression matrix was normalized using NormalizeData function, followed by selecting top 2,000 most variable genes across datasets using FindVariableFeatures and SelectIntegrationFeatures functions. To correct the batch effect, FindIntegrrationAnchors and IntegrateData functions were used based on the selected feature genes. The corrected dataset was subjected to standard Seurat workflow for dimension reduction and clustering. In this study, clusters of therapeutic cells were manually merged and annotated based on gene signatures reported from Human Protein Atlas (proteinatlas.org) and previous studies, and clusters of mouse immune cells were merged and annotated based on the immune lineage markers. AddModuleScore was used to calculate module scores of each list of gene signatures, and FeaturePlot function was used to visualize the expression of each signature in the UMAP plots. For gene set enrichment analysis (GSEA), clusterProfiler packages were used to calculate the enrichment scores of each cluster in the signature gene list.

### *In vitro* tumor cell killing assay

Tumor cells (1 × 10^4^ cells per well) were co-cultured with effector cells (at ratios indicated in the figure legends) in C10 medium in Corning 96-well clear-bottom black plates for 24 hours. At the end of the culture, live tumor cells were quantified by adding D-luciferin (150 μg/mL; Fisher Scientific, cat. no. 50-209-8110) to the cell cultures and measuring luciferase activity using an Infinite M1000 microplate reader (Tecan).

### *In vivo* bioluminescence live animal imaging (BLI)

BLI was performed using a Spectral Advanced Molecular Imaging (AMI) HTX imaging system (Spectral instrument Imaging). Live animal images were acquired 5 min after intraperitoneal (i.p.) injection of D-luciferin for total body bioluminescence. A dose of 1 mg per mouse was administered to monitor the signal of tumor cells (i.e., Raji-FG cells), while 3 mg per mouse was injected to monitor therapeutic cells (i.e., CAR-iNKT-FG cells). Imaging data were analyzed using AURA imaging software (Spectral Instrument Imaging, v.3.2.0).

### *In vivo* PK/PD study: Human melanoma xenograft NSG mouse model

On day 0, NSG mice received subcutaneous (s.c.) inoculation of A375 human melanoma cells (1 × 10^6^ cells into both left and right sides per mouse). On day 4, the experimental mice received paratumoral (p.t.) implantation of iMRAS on the left side only. On day 5, the experimental mice received intravenous (i.v.) injection of CAR-iNKT-FG cells (5 × 10^6^ cells in 100 μl of PBS per mouse). Over the experiment, mice were monitored for survival, and iNKT-FG cells were measured using BLI.

### *In vivo* antitumor efficacy study: Human melanoma xenograft NSG mouse model

On day 0, NSG mice received s.c. inoculation of A375-CD19 human melanoma cells (1 × 10^6^ cells per mouse). On day 4, the experimental mice received either no treatment or p.t. implantation of iMRAS at the tumor site. On day 5, the experimental mice received i.v. injection of vehicle (100 μl PBS per mouse), CAR-iNKT cells (5 × 10^6^ CAR+ cells in 100 μl PBS per mouse), or CAR-T cells (5 × 10^6^ CAR^+^ cells in 100 μl PBS per mouse). Over the experiment, mice were monitored for survival and tumor volume. Tumor weight was measured on the terminal day, Day 30.

### *In vivo* antitumor efficacy study: Human lymphoma xenograft NSG mouse model

On day 0, NSG mice received i.v. inoculation of Raji-FG human B cell lymphoma cells (2 × 10^5^ cells per mouse). On day 4, the experimental mice received either no treatment or p.t. implantation of iMRAS. On day 5, the experimental mice received i.v. injection of vehicle (100 μl PBS per mouse), CAR-iNKT cells (5 × 10^6^ CAR^+^ cells in 100 μl PBS per mouse), or CAR-T cells (5 × 10^6^ CAR^+^ cells in 100 μl PBS per mouse). Over the experiment, mice were monitored for survival, and their tumor loads were measured using BLI.

### Histology analysis

Tissues (e.g., bone, spleen, liver, and lung) were collected from experimental mice, fixed in Epredia^™^ 10% Neutral Buffered Formalin (Fisher Scientific; cat. no. 22-050-105) for up to 36 hours and embedded in paraffin for sectioning (4 μm thickness). For bone samples, the bones were treated with hydrochloric acid (Fisher Scientific; cat. no. SA49) to achieve decalcification. Tissue sections were prepared and stained with hematoxylin and eosin by the UCLA Translational Pathology Core Laboratory, following the Core’s standard protocols. Stained sections were imaged using an Olympus BX51 upright microscope equipped with an Optronics Macrofire CCD camera (AU Optronics). The images were analyzed using Optronics PictureFrame software (AU Optronics).

### Statistics

Graphpad Prism v.8 software (Graphpad) was used for statistical data analysis. Student’s two-tailed t-test was used for pairwise comparisons. Ordinary one-way analysis of variance (ANOVA) followed by Tukey’s or Dunnett’s multiple comparisons test was used for multiple comparisons. A log rank (Mantel–Cox) test adjusted for multiple comparisons was used for Meier survival curves analysis. Data are presented as the mean ± SEM, unless otherwise indicated. In all figures and figure legends, n represents the number of samples or animals used in the indicated experiments. A *P* value of less than 0.05 was considered significant. ns, not significant; *p < 0.05, **p < 0.01, ***p < 0.001, ****p < 0.0001.

## Data Availability

The scRNA-seq data generated in this study have been deposited in the public repository Gene Expression Omnibus Database: GSE291443. The remaining data are available within the Article, Supplementary Information or Source Data file.
